# Evidence Regarding Automatic Processing Computerized Tasks Designed For Health Interventions in Real-World Settings Among Adults: Systematic Scoping Review

**DOI:** 10.2196/17915

**Published:** 2020-07-29

**Authors:** Harshani Jayasinghe, Camille E Short, Annette Braunack-Mayer, Ashley Merkin, Clare Hume

**Affiliations:** 1 School of Public Health The University of Adelaide Adelaide Australia; 2 Melbourne Centre for Beahviour Change, Melbourne School of Psychological Science and Melbourne School of Health Science University of Melbourne Melbourne Australia; 3 School of Health and Society University of Wollongong Wollongong Australia; 4 School of Medicine The University of Adelaide Adelaide Australia

**Keywords:** automatic processing, computerized tasks, health interventions, real-world, adult, behavior change, mobile phone

## Abstract

**Background:**

Dual process theories propose that the brain uses 2 types of thinking to influence behavior: automatic processing and reflective processing. Automatic processing is fast, immediate, nonconscious, and unintentional, whereas reflective processing focuses on logical reasoning, and it is slow, step by step, and intentional. Most digital psychological health interventions tend to solely target the reflective system, although the automatic processing pathway can have strong influences on behavior. Laboratory-based research has highlighted that automatic processing tasks can create behavior change; however, there are substantial gaps in the field on the design, implementation, and delivery of automatic processing tasks in real-world settings. It is important to identify and summarize the existing literature in this area to inform the translation of laboratory-based research to real-world settings.

**Objective:**

This scoping review aims to explore the effectiveness of automatic training tasks, types of training tasks commonly used, mode of delivery, and impacts of gamification on automatic processing tasks designed for digital psychological health interventions in real-world settings among adults.

**Methods:**

The scoping review methodology proposed by Arskey and O’Malley and Colquhoun was applied. A scoping review was chosen because of the novelty of the digital automatic processing field and to encompass a broad review of the existing evidence base. Electronic databases and gray literature databases were searched with the search terms “automatic processing,” “computerised technologies,” “health intervention,” “real-world,” and “adults” and synonyms of these words. The search was up to date until September 2018. A manual search was also completed on the reference lists of the included studies.

**Results:**

A total of 14 studies met all inclusion criteria. There was a wide variety of health conditions targeted, with the most prevalent being alcohol abuse followed by social anxiety. Attention bias modification tasks were the most prevalent type of automatic processing task, and the majority of tasks were most commonly delivered over the web via a personal computer. Of the 14 studies included in the review, 8 demonstrated significant changes to automatic processes and 4 demonstrated significant behavioral changes as a result of changed automatic processes.

**Conclusions:**

This is the first review to synthesize the evidence on automatic processing tasks in real-world settings targeting adults. This review has highlighted promising, albeit limited, research demonstrating that automatic processing tasks may be used effectively in a real-world setting to influence behavior change.

## Introduction

### Background

Digital psychological health interventions, which encompass both behavior change and mental health interventions, are increasingly being adopted to prevent and manage chronic health conditions. This includes stroke [1], dementia [2], obesity [3], addiction [4], and mood disorder [5]. The effectiveness of these interventions may be increased by the adoption of dual process theories that propose that the brain uses 2 types of processes to influence behavior [6-8]: automatic processing and reflective processing.

Automatic processing is fast, immediate, unconscious, and unintentional [9,10]. It involves an appraisal of a stimulus (eg, a basketball) in terms of its affective and conditioned properties. A mental representation of the stimulus and the network of associated concepts is automatically activated. By activating these mental networks, stimuli can capture or repel attention (attentional bias), lead to an automatic judgment of the stimulus as good or bad (automatic evaluations), and elicit a tendency to approach or avoid the stimulus (approach or avoidance bias). Cognitive biases are mostly automatic processes that result in individuals giving increased attention to threatening stimuli, with the difficulties in disengaging from these stimuli [11]. In contrast, reflective processing focuses on logical reasoning; it is slow, step by step, voluntary, and intentional, such as self-regulatory processes [9,10]. These 2 types of processes can occur simultaneously and can have concordant or opposing influences on behavior [10]. For example, when a person sees a basketball, this will trigger a network of concepts in the procedural memory, such as *fun* and *good* or *hard* and *bad*, which can lead to an automatic response from the individual to either approach or avoid the basketball (approach-avoidance bias). If the stimuli are automatically perceived as positive, it will result in an approach response, whereas if the stimuli are automatically perceived as negative, it will result in an avoidance response [12,13]. In this instance, these automatic associations could influence the formation of intentions to play basketball in the first place or may contribute to an intention-behavior gap if the conscious, logical intention is at odds with the automatic association.

Given that both types of processes can occur simultaneously and that behavior change may be most likely when they are congruent, the dual process theory suggests that both the reflective and automatic processing pathways should be targeted to create and sustain behavior change [8,14]. However, most digital behavior change and mental health interventions tend to target the reflective system only, aiming to improve self-regulatory processes, for example, processes such as self-monitoring behavior, goal-setting, or cognitive behavioral therapy [10,15]. Although these strategies are useful for supporting intention or goal-directed behavior, there is also clear evidence of an intention-behavior gap [16].

Laboratory-based evidence suggests that digital interventions delivered via computerized tasks can change people’s automatic processes to specific stimuli [14,17-19]. Automatic processes can be retrained using cognitive bias modifications (CBM) tasks, attentional bias modification tasks, and evaluative conditioning tasks [14,18,20-22]. CBM tasks have been extensively tested in the field of anxiety and depression in laboratory settings [14,18,20-22] and work by targeting attentional and interpretative biases away from threats [14]. The dot-probe test is often used to assess attentional biases [23] and involves the presentation of pairs of stimuli, one of which is threatening and one that is neutral. Participants are shown the stimuli simultaneously, one stimulus on either end of a computer screen, for a small amount of time (eg, 500 ms); a dot then appears in the place of one former stimulus, and participants are asked to indicate the location of this dot as quickly as possible. The computer automatically measures the speed of this reaction. The test is then repeated several times. Quicker response times to the dot when it appears in the location of threatening stimuli are interpreted as attentiveness to threat [23]. Dot-probe tasks can also be used to retrain attentional biases by replacing a targeted probe (designed to change attentional biases) with neutral or salient stimuli during all cycles of the task [19,20,24-26]. A commonly used task shows participants a smiling or neutral face alongside an unhappy or angry face; participants are then asked to select the positive image as fast as possible [14]. Through the repetitive nature of the task, a person’s automatic processes can be reconditioned toward a particular stimulus [14].

Common criticisms of automatic processing tasks are that they are repetitive and boring [18,27]. Recently, the field has attempted to make these tasks more enjoyable and accessible by delivering these tasks on digitally ubiquitous technology such as smartphone apps and drawing on the developing realm of gamification. Gamification seeks to motivate and engage users by using gaming style elements often seen in games (rewards, points, and leader boards). A review by Boendermaker et al [19] into gamified CBM highlighted that there were many projects currently in progress; however, there still remained a lack of evidence to draw any firm conclusions regarding the effectiveness of gamification on increasing engagement. Similarly, Zhang et al [11] highlighted that understanding gamification approaches is crucial in future conceptualization and codesign of attention bias modification interventions.

### Objectives

Despite the evidence of the importance of the automatic processing pathway for regulating behavior, the majority of reviews on automatic processing tasks have been limited, focusing mainly on mental health conditions delivered in laboratory settings [17,18,28-30]. In addition, existing reviews have routinely included studies with children whose brain development differs from that of adults [31-33]; thus, a need to map the existing literature examining automatic processing pathways in digital health interventions in real-world settings among adult populations was identified [16,27,34,35]. This scoping review aimed to explore the effectiveness of automatic training tasks, types of training tasks commonly used, mode of delivery, and impacts of gamification on automatic processing tasks designed for digital psychological health interventions in real-world settings among adults.

## Methods

### Scoping Review Methodology

Scoping reviews aim to “map rapidly the key concepts underpinning a research area and the main sources and types of evidence available” [36]. This scoping review sought to address the question: “what is the current evidence base around design and effectiveness of automatic processing computerized tasks designed for health interventions in real-world settings among adults?” A scoping review was chosen because of the novelty of the digital automatic processing field and to encompass a broad review of the evidence. Scoping reviews allow the development of inclusion and exclusion criteria during the study selection, the inclusion of all types of studies, and extraction of data regarding key issues and themes, in contrast to systematic reviews that are much more stringent with synthesis [37,38].

This scoping review followed the framework described by Arksey and O’Malley [38], who provided a detailed description of how to conduct a methodologically rigorous scoping review. In addition, the current best practices of Colquhoun et al [37] for the conduct of scoping reviews’ guidelines were also applied. Arksey and O’Malley’s methodology [38] comprised the following steps: (1) identifying the research question; (2) identifying relevant studies; (3) study selection; (4) charting the data; and (5) collating, summarizing, and reporting the results. A number of additional recommendations from a study by Colquhoun et al [37] were also integrated: development of a protocol before the initial scoping study began, using Preferred Reporting Items for Systematic Review and Meta-Analysis (PRISMA) Protocols, the use of 2 independent reviewers, use of same inclusion criteria during initial data screening, and full-text screening and pilot testing of the data extraction template.

### Identifying Relevant Studies

#### Search Strategy

A systematic literature search was created with the assistance of an academic librarian from the University of Adelaide. It was applied to all databases by HJ. The search was completed using the electronic databases PubMed, Scopus, Excerpta Medica Database (EMBASE), Psychological Information Database (PsycINFO), Web of Science, Cumulative Index to Nursing and Allied Health Literature (CINAHL), Cochrane Database of Systematic Reviews, and Google Scholar. The search was performed in September 2018. A gray literature search was also conducted to identify any published or unpublished data not found through the initial search. This included electronically searching the repositories holding theses and research papers: Trove, The Gray Literature Report, ProQuest, OpenGrey, and Grey Literature Network Service (GreyNet International). Reference lists of all included studies were manually screened to identify possible relevant citations. The search strategy created was based on 5 main components: (1) automatic processing, (2) computerized tasks, (3) health interventions, (4) real world, and (5) adult. Relevant keywords were identified using Medical Subjects Headings (MeSH) and Emtree terms, synonyms, and keywords from relevant articles (Multimedia Appendix 1).

The results of the search were uploaded into EndNote X 7.3.1 (Clarivate Analytics), and duplicates were then removed and exported into Rayyan. Rayyan is a web-based tool that assists in the completion of systematic reviews. Rayyan was initially used by HJ to screen titles search and determine eligibility. This was followed by an abstract screen using the inclusion and exclusion criteria. If eligibility was ambiguous, criteria were discussed with other coauthors until consensus was reached. An eligibility proforma was also used during this process. Articles matching the inclusion criteria were then selected for full-text analysis.

#### Inclusion and Exclusion Criteria

Inclusion criteria for the study were adults aged older than 18 years, designed to be a health intervention (defined as aiming to improve physical or mental health), delivered via a computerized (digital) task, and delivered in a real-world setting (a free-living environment, excluding a laboratory-based environment). Only studies published in English between the years 2000 and 2018 were included. Articles not published in English were excluded. Articles were also excluded if they were published before the year 2000 because of digital expansion in the field of automatic processing predominately occurring after the year 2000.

#### Charting the Data

Data extraction was performed using a standardized data extraction template with the following fields: author, year, country, aims of the study, setting and population, participant demographics, details of the intervention and comparators, study methodology, sampling and recruitment, completion rates, and intervention details (Multimedia Appendix 2). HJ, CES, CH, and AB all conducted data extraction for the first 2 publications using the original data extraction template. All reviewers then discussed any iterations, and the template was changed appropriately to reflect any inconsistencies. Data for all remaining studies were extracted by HJ, with 10% being verified by AM. Conflicts or concerns during this period were resolved through discussion with CES and CH.

#### Collating, Summarizing, and Reporting the Results

The data extraction forms were used to form quick overview summaries of the included studies. A descriptive numerical summary was used to create a numerical overview of general study characteristics, and then a narrative overview was conducted on the type of automatic processing task used and effectiveness.

## Results

### PRISMA Results

**Figure 1:**
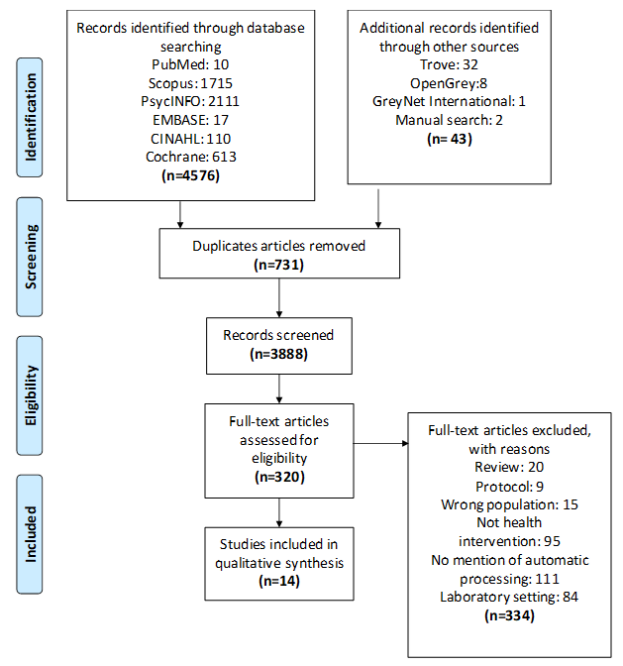
Preferred Reporting Items for Systematic Reviews and Meta-Analyses for the results of the search. CINAHL: Cumulative Index of Nursing and Allied Health Literature; EMBASE: Excerpta Medica Database; GreyNet: Grey Literature Network Service; PsycINFO: Psychological Information Database.

The PRISMA flow chart for the results of the search is presented in Figure 1. The flowchart contains results from the initial search, how many studies were removed because of duplications, study selection, and amount selected for full-text analysis (Figure 1). The search is up to date until September 2018; 4576 studies were found to be eligible for inclusion (Figure 1). Of these, 320 were assessed for full-text inclusion, of which 14 studies met all inclusion criteria. The reasons for study exclusion included being a review paper, irrelevant to the topic, and not being set in a real-world setting.

### General Characteristics

Characteristics including the type of study, sample size, country, and follow-up are reported in Table 1. Most of the included studies were conducted in Europe and the United States, with 6 in Europe and 5 in the United States. One study was conducted in China [39] and another in Australia [34]. All studies were randomized controlled trials, except one that was a cross-sectional study. Sample sizes ranged from 21 to 672 participants. Five of the studies targeted automatic processes regarding alcoholism [19,20,31,32,35]. Two studies targeted smoking attitudes and behavior [35,40]. Two studies targeted automatic processes toward anxiety [21,39], and one study targeted automatic processing toward both anxiety and depression [20]. In addition, one study was found for insomnia [34], self-injurious thoughts and behaviors [41], attitudes toward homosexuality and self-esteem in gay men [42], and relationship satisfaction in marriages [43]. Follow-up periods also varied and ranged from no follow-up (n=4 studies [39,42,44,45]) to the longest follow-up period of 6 months [35].

**Table 1:** General demographics of included studies (n=14).

Reference	Type of study	Population size (N)	Country	Follow-up
Robinson et al (2017) [40]	RCT^a^	64	The United States	Two weekly follow-ups
Clarke et al (2016) [34]	RCT	36	Australia	8 days
Yang et al (2017) [39]	RCT	76	China	No follow-up
Fleming and Burns (2017) [42]	RCT	274	The United States	No follow-up
van Deursen et al (2015) [45]	Cross-sectional	437	Germany	No follow-up
de Voogd et al (2016) [20]	RCT	340	The Netherlands	Baseline measures were taken before training and then 3, 6, and 12 months after training
Enock et al (2016) [21]	RCT	429	The United States	Pretest, posttest, and 1- and 2-month follow-ups
Monk et al (2017) [44]	RCT	62	The United Kingdom	No follow-up
Wiers et al (2015) [26]	RCT	615	The Netherlands	The first follow-up was 1 month after the posttest (N=109, 35.0%) and the second 2 months later (N=87, 28.0%)
Boendermaker et al (2016) [25]	RCT	96	The Netherlands	Four sessions conducted at least one day apart and 2-week follow-up after session 4
Crane et al (2018) [46]	RCT	672	The United Kingdom	The follow-up questionnaire was sent to participants 28 days after downloading the app
Elfeddali et al (2016) [35]	RCT	475	Amsterdam	A posttraining assessment (ie, manikin and visual probe tasks) followed the intervention sessions. An assessment of continued smoking abstinence followed 6 months after baseline
Franklin et al (2016) [41]	RCT	114, 131, and 163	The United States	1-month follow-up
McNulty et al (2017) [43]	RCT	288	The United States	2 weeks

**^a^**RCT: randomized controlled trial.

### Intervention Characteristics

#### Types of Digital Technology

Table 2 reports the intervention characteristics of the included studies. The type of digital technology most often used to execute the intervention was a personal computer or laptop via a website (n=8) [20,25,26,35,42-45], followed by the use of a smartphone app (n=5) [21,34,39,41,46]. One study was delivered on a personal digital assistant device [40].

**Table 2:** Intervention characteristics of included studies (n=14).

Reference	Type of digital technology	Real-world setting
Robinson et al (2017) [40]	Hewlett-Packard iPAQ Personal Digital Assistant	Laboratory and home
Clarke et al (2016) [34]	Smartphone	Laboratory and home
Yang et al (2017) [39]	Smartphone	Home
Fleming and Burns (2017) [42]	Functioned only on desktop or laptop personal computers	Web-based
van Deursen et al (2015) [45]	Personal computer or laptop	Web-based
de Voogd et al (2016) [20]	Computer	Web-based at school and home
Enock et al (2014) [21]	Smartphone	Web-based
Monk et al (2017) [44]	Computer, mobile prohibited	Web-based
Wiers et al (2015) [26]	Computer	Web-based
Boendermaker et al (2016) [25]	Computer	Laboratory and home
Crane et al (2016) [46]	Smartphone app	Web-based
Elfeddali et al (2016) [35]	Computer	Web-based
Franklin et al (2016) [41]	Mobile app but can be used on phones, tablets, laptops, and desktops	Web-based
McNulty et al (2017) [43]	Computer	Laboratory and home

#### Use of Dual Process Theory

A total of 5 of the 14 included studies referred to dual process theory when explaining the theory behind the use of bias modification tasks [25,26,35,45,46]. Moreover, 7 studies did not mention any theories guiding their research [20,21,34,39-41,44]. Furthermore, 2 studies did not mention dual process theories but referred to other theories as a guide to their research [42,43].

Only 2 of the included studies [45,46] focused their interventions on targeting both parts of the dual process theory, targeting both automatic processing and reflective processing pathways. Crane et al [46] evaluated module components of an alcohol reduction app called *Drink Less* to change participants’ drinking behavior in a factorial trial. Of the 5 modules tested, 1 (cognitive bias retraining module) targeted automatic processes regarding the impulse to drink alcohol. There was a significant two-way interaction between theis module on weekly alcohol consumption (*P*=.03), indicating that enhanced normative feedback (targeting misperceptions via reflective processes) led to a significant reduction in weekly alcohol consumption only when combined with enhanced cognitive bias retraining. van Deursen et al [45] examined the relationship among problem drinkers seeking web-based help to change their alcohol use, hypothesizing that executive functions would moderate the relationship between automatic associations and drinking and that this effect would be stronger in individuals with strong motivation to change. A brief *Implicit Association Test* was used to test valence and approach associations, whereas executive (reflective) pathways were assessed via a number of smaller tests. The study results provided partial support for the moderating role of motivation in the interplay between automatic processes and executive functions.

#### Types of Automatic Processing Tasks

Three types of automatic processing tasks were used in the included studies: attentional bias modification tasks, CBM tasks, and evaluative conditioning tasks. Attentional bias modification tasks were the most commonly delivered intervention through the use of dot-probe, visual probe, and visual search tasks. CBM tasks were delivered via dot-probe test [39], word sentence association paradigms [39], attentional control training, approach bias retraining [26], and cognitive bias retraining [46]. Evaluative conditioning was delivered via evaluative conditioning tasks [42,43], a stop-signal task, and a game-like therapeutic evaluative conditioning task.

#### Usage

Intervention durations varied considerably between the studies that reported on how long the intervention was delivered for, ranging from the shortest intervention period of 75 min to the longest intervention period of 6 weeks. All studies delivered at least some of their interventions in a real-world setting (as per inclusion criteria), with 4 studies delivering interventions in a mixed setting (both laboratory and home environments). Some studies have reported problems with usage. This included the number of training trials used over sessions being too low, preventing the training from potentially changing attentional biases [25]; low training compliance, with some participants dropping out of the study midway effecting overall results and limiting generalizability of findings [20,26,40,46]; and having self-selected use and dosage during the intervention, making it hard to determine the optimal dosage needed for intervention [41].

#### Gamification Strategies

Three of the included studies incorporated elements of gamification to enhance engagement of their intervention [25,41,46]. Boendermaker et al gamified [25] an existing automatic processing task, which aimed to train attention away from pictures of alcoholic beverages via a visual probe task. They found that the gamified automatic processing task did not improve participant motivation to train as compared with the usual ungamified task. In fact, some aspects of motivation appeared to deteriorate rather than improve. Crane et al [46] and Franklin et al [41] saw positive effects with the use of gamified elements in their interventions. However, the effect that gamification had on influencing study outcomes was not investigated, making it difficult to deduce which aspects of gamification produced different effects and overall how effective gamification was.

#### Outcome Assessments

Two studies reported that it was possible that the option to train at home had a negative effect on the final results, as it may have made participants take the training less seriously. It was also noted that the web-based nature of web-based assessments resulted in issues with standardization of interventions, making it hard to control how measures were completed [45]. The large number of measures included in some studies may have also resulted in participant fatigue [45], and the use of similar types of tasks testing different outcomes may have resulted in practice effects that may have affected the overall findings.

#### Effectiveness of Interventions

A detailed description of the aims, measures, and effectiveness of included studies can be found in Multimedia Appendix 3 [14,15,19,20,29-34,36-39]. A total of 14 studies assessed changes in automatic processes, whereas 11 studies assessed how changes in automatic processing contributed to changes in behavior. Of the 14 studies that assessed changes in automatic processes, 8 reported a statistically significant effect in the direction expected [21,25,26,34,40,43,44,46]. Of the remaining 6 studies evaluating automatic processes, 4 reported no significant effects on changes in automatic processes [20,35,42,45].

Of the 11 studies that assessed behavioral or mental health outcomes, 4 reported a significant intervention effect [21,26,34,43] and 3 of these included automatic processing intervention strategies only. Five others reported on behavioral or mental outcomes but saw no significant results on changes to outcomes [20,25,35,42,46].

Three studies reported mixed findings [39-41] on changes to automatic processes, with 2 of the studies also further detailing changes to behavior. Robinson et al [40] found that a mobile-delivered attentional bias intervention could reduce attentional bias toward thoughts about smoking but had mixed effects on changing smoking behavior. It was thought that these findings may have been impacted by participant attrition affecting overall results and the sample being nontreatment seeking participants [40]. Yang et al found [39] that 1 of the 3 CBM tools assessed could be used effectively to reduce anxiety and mood problems. However, the other 2 methods assessed yielded limited effectiveness. The authors reported that a low sample size and a lack of engagement elements to make the tasks *fun* may have impacted the results [39]. Franklin et al [41] conducted 3 evaluative conditioning studies designed to reduce self-injurious thoughts and behaviors, finding that 2 of the studies successfully reduced self-injurious thoughts and behaviors. It was suggested that identifying additional treatment targets, such as other self-injurious thoughts and behaviors not covered by the study, and increasing digital engagement strategies for users may yield better results across all 3 studies in the future [41].

Some of the authors of the studies that showed no significant effects on changes in automatic processes to behaviors outlined possible reasons within their manuscripts. Boendermaker et al [25] attributed these findings to small attentional biases at baseline and low numbers of training trials as compared with other trials in the areas affecting dose-response relationships and having a web-based intervention in a real-world setting, which may have impacted participants’ motivation by making them take the training less seriously. Fleming and Burns [42] attributed the null findings to having biased unrepresentative sample populations and the web-based nature of the intervention being available only via a personal computer or laptop limiting those who had an affinity for mobile use. de Voogd et al [20] inferred that the negative findings may have been because of participant dropout, as most adolescents did not complete all 8 intended training sessions. Elfeddali et al [35], Boendermaker et al [25], and de Voogd et al [20] also highlighted that motivation for web-based training appeared low, which they partly attributed to the repetitive nature of the training tasks and the web-based nature of the training tasks, which were completed at home and resulted in a lack of supervision or standardization of training circumstances.

## Discussion

### Principal Findings

This scoping review aimed to explore the effectiveness of automatic training tasks, types of training tasks commonly used, mode of delivery, and impacts of gamification on automatic processing tasks designed for digital psychological health interventions in real-world settings among adults. A small but developing evidence base was found. Of the 14 studies reviewed, only under half of the interventions resulted in positive changes to automatic processes. The positive trials provide some evidence that this approach may be possible in the real world, although many trials produced mixed results and issues with compliance and engagement were commonly described.

### Types of Training Tasks

The review identified 2 main types of tasks commonly used in the field to change automatic processing: attentional bias modification tasks and CBM tasks. These tasks can be delivered via a variety of methods, but the most popular in the health domain are dot-probe, visual probe, and visual search tasks. All 3 tasks have been extensively used and reviewed in the literature [19,47,48]; however, historically, these tasks have been used in a laboratory setting, particularly in mental health interventions seeking to change anxiety and depression. This review revealed an expansion in the field, both in a real-world setting and in other health fields such as problem drinking, smoking, and suicide.

### Usability

In the last few years, there has been a shift toward the use of smartphone apps for the delivery of automatic processing interventions. All automatic processing tasks contained within this review were deployed over the web, which made them easily accessible for use in the real world. They were most commonly delivered via a computer or laptop, with over half of the studies using this as the mode of delivery. This aligns with other reviews in the field, such as a recent review of attention and CBM apps by Zhang et al [11], which found 24 CBM apps that were commercially available. Although app usage is increasing, Zhang et al [11] found that most apps (n=8) were not rigorously evaluated, whereas the other 17 were all commercial apps, of which only one was evaluated in published literature. This review has been able to add to the work by Zhang et al [11], who found that 5 smartphone-based CBM task studies have been scientifically evaluated.

Digital advancements are increasingly facilitating pathways into real-world investigations in this field. Although digital platforms do increase participant accessibility in real-world environments, there are limitations to this approach. Three studies noted that the web-based nature of tasks, the lack of supervision, and standardization from external distractors may have negatively impacted the results [20,25,35]. Wiers et al [26] highlighted that this may have been because of large dropout rates commonly seen in web-based experiments and suggested making interventions more engaging to combat this. Boendermaker et al [25] and Elfeddali et al [35] suggested that allowing participants to do the training part of the intervention at home may have affected their motivation levels by them taking the task less seriously. de Voogd et al [20] proposed that the mixed results in that study may have been because of the lack of *stress* imposed by the laboratory environment, where most studies in this area have been conducted traditionally. Indeed, the stress of laboratory environments may, in fact, be beneficial to study outcomes, as participants may have taken the training task less seriously in their home environment, thereby negatively affecting conditioning effects. A review by Santarossa et al [49] in the field of health behavior change also shows that digital interventions are more effective when they have a human support element.

### Effectiveness of Changing Automatic Processes

Similar to other reviews in the area [32,33], this review also found mixed findings on the effectiveness of automatic processing tasks in real-world conditions. Of the effective interventions, over half of the studies targeted changing automatic processes toward alcohol. The successful characteristics of these interventions included the use of evaluative conditioning or CBM tasks for intervention delivery, the use of personal computers for mode of delivery, and the use of both elements of the dual process theory.

Mixed results on effectiveness were found for smoking, social anxiety, and self-injurious thoughts and behaviors. This may have been because of participant attrition affecting overall results, and Franklin et al [41] suggested that mixed findings in that study may have been because of issues with a lack of engagement elements. This is a common criticism of bias modification tasks, as they are often reported to be quite boring and repetitive by participants. There has been development in the field to make these tasks more engaging by adding elements of gamification that use visuals, sound effects, point systems, and rewards to make the tasks more engaging [19,24-27,50]. Three of the included studies incorporated elements of gamification to enhance engagement of their interventions [25,41,46]. Boendermaker et al [25] gamified an existing automatic processing task that aimed to train attention away from pictures of alcoholic beverages via a visual probe task. They found that the gamified automatic processing task did not improve participants’ motivation to train as compared with the usual nongamified task. In fact, some aspects of motivation appeared to deteriorate rather than improve, suggesting that gamification could have drawbacks if not done optimally.

Crane et al [46] and Franklin et al [41] observed positive effects of study outcomes with the use of games and gamified elements in their interventions. However, the effect that gamification had on influencing outcomes was not investigated. Other reviews have similarly found mixed findings with the use of gamification in automatic and reflective processing interventions [11,19,26,51].

### Strengths and Limitations

To our knowledge, this is the first study in the field to review automatic processing studies that specifically focus on real-world settings and adults. The expansive search that included both database and gray literature searching was a strength of this study. This allowed an extensive gathering of evidence to map key concepts and ideas in the field currently.

The eligibility criteria may have limited findings from key studies that did not meet the eligibility requirements, for example, many papers in the field that focus on evaluating automatic processing tasks in real-world settings among children, which may collectively hold key insights into the field at large [24,31,52]. In addition, following the best practice recommendations of conducting scoping reviews from Colquohon et al [37] and Arksey and O’Malley [38], included studies were not assessed for quality in relation to areas of bias such as randomization. Scoping reviews provide a breadth of information rather than the assessment of quality. The disadvantage of this is that it makes it difficult to gain an insight into the robustness and generalizability of the findings. However, the benefit of this method is that it allows the mapping of a wider range of available resources, painting an overall picture of the field at large, as the guidelines for inclusion are not as stringent as a systematic review. The results of a scoping review can, however, sometimes inform the development of a systematic review, which is better placed to deliver an assessment of quality. Finally, only primary studies published in English were included, resulting in a small number of studies for inclusion, which is a common limitation in scoping review [53].

### Future Directions

The increasing use of digital platforms to deliver automatic processing tasks, while increasing population reach and accessibility, does have drawbacks. Although monitoring and standardization levels are relatively achievable in laboratory-based environments, it is often difficult to monitor compliance and ensure adherence in real-world studies. Future studies could experiment with different instructions or persuasion techniques for completing the training as well as different training paradigms to increase compliance. Furthermore, there may be concerns about privacy and confidentiality issues and require further research [54]. Engagement of automatic processing tasks remains a prominent issue because of their inherent boring nature. Gamification offers promising capabilities, and future research should further investigate how its incorporation can enhance enjoyment in the field. Although research into gamification is mixed, studies contained within this review have highlighted gamification as an important engagement strategy [20,26,35], which if implemented correctly could enhance the enjoyment of traditionally mundane tasks. Finally, it was unfortunate that there were few studies in the field that targeted both processes during their interventions; both processes alone have shown significant ability to change behavior, and combining these processes could improve the design and effectiveness of future health interventions and could be a crucial missing link.

### Conclusions

This is the first review to synthesize the evidence for published and gray literature on automatic processing tasks set in real-world settings targeting adults. This review has highlighted promising, albeit limited, research demonstrating that automatic processing tasks may be used effectively in a real-world setting to influence behavior change. Given that several trials with negative findings were also identified, future research is needed to understand why significant effects are observed in some contexts and not others and how to optimize delivery for optimal engagement and efficacy.

## Appendix

Multimedia Appendix 1 Search strategy.

Multimedia Appendix 2 Data extraction template.

Multimedia Appendix 3 Automatic processing targeted health outcomes and effectiveness.

## Abbreviations

CBM: cognitive bias modification
CINAHL: Cumulative Index of Nursing and Allied Health Literature
EMBASE: Excerpta Medica Database
GreyNet: Grey Literature Network Service
PRISMA: Preferred Reporting Items for Systematic Reviews and Meta-Analysis
PsycINFO: Psychological Information Database
RCT: randomized controlled trial

## References

[1] Zhang MW, Ho RC (2016). Harnessing the potential of the Kinect sensor for psychiatric rehabilitation for stroke survivors. Technol Health Care.

[2] Zhang MW, Ho RC (2017). Personalized reminiscence therapy m-health application for patients living with dementia: innovating using open source code repository. Technol Health Care.

[3] Zhang MW, Ho RC, Cassin SE, Hawa R, Sockalingam S (2015). Online and smartphone based cognitive behavioral therapy for bariatric surgery patients: initial pilot study. Technol Health Care.

[4] Zhang MW, Ho RC (2016). Tapping onto the potential of smartphone applications for psycho-education and early intervention in addictions. Front Psychiatry.

[5] Rajagopalan A, Shah P, Zhang MW, Ho RC (2017). Digital platforms in the assessment and monitoring of patients with bipolar disorder. Brain Sci.

[6] Corr PJ (2010). Automatic and controlled processes in behavioural control: implications for personality psychology. Eur J Pers.

[7] Carver CS, Johnson SL, Joormann J (2008). Serotonergic function, two-mode models of self-regulation, and vulnerability to depression: what depression has in common with impulsive aggression. Psychol Bull.

[8] Schneider W (2003). Controlled & automatic processing: behavior, theory, and biological mechanisms. Cogn Sci.

[9] Strack F, Deutsch R (2004). Reflective and impulsive determinants of social behavior. Pers Soc Psychol Rev.

[10] Cheval B, Radel R, Neva JL, Boyd LA, Swinnen SP, Sander D, Boisgontier MP (2018). Digital platforms in the assessment and monitoring of patients with bipolar disorderbehavioral and neural evidence of the rewarding value of exercise behaviors: a systematic review. Sports Med.

[11] Zhang MW, Ying JB, Song G, Ho RC (2018). A review of gamification approaches in commercial cognitive bias modification gaming applications. Technol Health Care.

[12] Conroy DE, Berry TR (2017). Automatic affective evaluations of physical activity. Exerc Sport Sci Rev.

[13] Rebar AL, Schoeppe S, Alley SJ, Short CE, Dimmock JA, Jackson B, Conroy DE, Rhodes RE, Vandelanotte C (2016). Automatic evaluation stimuli - the most frequently used words to describe physical activity and the pleasantness of physical activity. Front Psychol.

[14] Beard C (2011). Cognitive bias modification for anxiety: current evidence and future directions. Expert Rev Neurother.

[15] Hofmann W, Friese M, Wiers RW (2008). Impulsive versus reflective influences on health behavior: a theoretical framework and empirical review. Health Psychol Rev.

[16] Rhodes RE, de Bruijn GJ (2013). How big is the physical activity intention-behaviour gap? A meta-analysis using the action control framework. Br J Health Psychol.

[17] Beard C, Sawyer AT, Hofmann SG (2012). Efficacy of attention bias modification using threat and appetitive stimuli: a meta-analytic review. Behav Ther.

[18] Beard C, Weisberg RB, Primack J (2012). Socially anxious primary care patients' attitudes toward cognitive bias modification (CBM): a qualitative study. Behav Cogn Psychother.

[19] Boendermaker WJ, Prins PJ, Wiers RW (2015). Cognitive bias modification for adolescents with substance use problems--can serious games help?. J Behav Ther Exp Psychiatry.

[20] de Voogd E, Wiers R, Prins P, de Jong P, Boendermaker W, Zwitser R, Salemink E (2016). Online attentional bias modification training targeting anxiety and depression in unselected adolescents: short- and long-term effects of a randomized controlled trial. Behav Res Ther.

[21] Enock PM, Hofmann SG, McNally RJ (2014). Attention bias modification training via smartphone to reduce social anxiety: a randomized, controlled multi-session experiment. Cogn Ther Res.

[22] Blankers M, Salemink E, Wiers R, Mucic D, Hilty DM (2016). Cognitive behavioural therapy and cognitive bias modification in internet-based interventions for mood, anxiety and substance use disorders. e-Mental Health.

[23] MacLeod C, Mathews A, Tata P (1986). Attentional bias in emotional disorders. J Abnorm Psychol.

[24] Boendermaker WJ, Boffo M, Wiers RW (2015). Exploring elements of fun to motivate youth to do cognitive bias modification. Games Health J.

[25] Boendermaker WJ, Sanchez Maceiras S, Boffo M, Wiers RW (2016). Attentional bias modification with serious game elements: evaluating the shots game. JMIR Serious Games.

[26] Wiers RW, Houben K, Fadardi JS, van Beek P, Rhemtulla M, Cox WM (2015). Alcohol cognitive bias modification training for problem drinkers over the web. Addict Behav.

[27] Lumsden J, Edwards EA, Lawrence NS, Coyle D, Munafò MR (2016). Gamification of cognitive assessment and cognitive training: a systematic review of applications and efficacy. JMIR Serious Games.

[28] Allen L, Mulgrew KE, Rune K, Allen A (2018). Attention bias for appearance words can be reduced in women: results from a single-session attention bias modification task. J Behav Ther Exp Psychiatry.

[29] Förderer S, Unkelbach C (2012). Hating the cute kitten or loving the aggressive pit-bull: EC effects depend on CS-US relations. Cogn Emot.

[30] Förderer S, Unkelbach C (2015). Attribute conditioning: changing attribute-assessments through mere pairings. Q J Exp Psychol (Hove).

[31] Lau JY (2015). Commentary: a glass half full or half empty? Cognitive bias modification for mental health problems in children and adolescents--reflections on the meta-analysis by Cristea et al (2015). J Child Psychol Psychiatry.

[32] Pennant ME, Loucas CE, Whittington C, Creswell C, Fonagy P, Fuggle P, Kelvin R, Naqvi S, Stockton S, Kendall T, Expert Advisory Group (2015). Computerised therapies for anxiety and depression in children and young people: a systematic review and meta-analysis. Behav Res Ther.

[33] Price RB, Wallace M, Kuckertz JM, Amir N, Graur S, Cummings L, Popa P, Carlbring P, Bar-Haim Y (2016). Pooled patient-level meta-analysis of children and adults completing a computer-based anxiety intervention targeting attentional bias. Clin Psychol Rev.

[34] Clarke PJ, Bedford K, Notebaert L, Bucks RS, Rudaizky D, Milkins BC, MacLeod C (2016). Assessing the therapeutic potential of targeted attentional bias modification for insomnia using smartphone delivery. Psychother Psychosom.

[35] Elfeddali I, de Vries H, Bolman C, Pronk T, Wiers RW (2016). A randomized controlled trial of web-based attentional bias modification to help smokers quit. Health Psychol.

[36] Dijkers M (2015). What is a Scoping Review?. KTDRR.

[37] Colquhoun HL, Levac D, O'Brien KK, Straus S, Tricco AC, Perrier L, Kastner M, Moher D (2014). Scoping reviews: time for clarity in definition, methods, and reporting. J Clin Epidemiol.

[38] Arksey H, O'Malley L (2005). Scoping studies: towards a methodological framework. Int J Soc Res Method.

[39] Yang R, Cui L, Li F, Xiao J, Zhang Q, Oei TP (2017). Effects of cognitive bias modification training via smartphones. Front Psychol.

[40] Robinson CD, Muench C, Brede E, Endrighi R, Szeto EH, Sells JR, Lammers JP, Okuyemi KS, Waters AJ (2017). Effect of attentional retraining on cognition, craving, and smoking in African American smokers. Psychol Addict Behav.

[41] Franklin JC, Fox KR, Franklin CR, Kleiman EM, Ribeiro JD, Jaroszewski AC, Hooley JM, Nock MK (2016). A brief mobile app reduces nonsuicidal and suicidal self-injury: evidence from three randomized controlled trials. J Consult Clin Psychol.

[42] Fleming JB, Burns MN (2017). Online evaluative conditioning did not alter internalized homonegativity or self-esteem in gay men. J Clin Psychol.

[43] McNulty JK, Olson MA, Jones RE, Acosta LM (2017). Automatic associations between one's partner and one's affect as the proximal mechanism of change in relationship satisfaction: evidence from evaluative conditioning. Psychol Sci.

[44] Monk RL, Qureshi A, Pennington CR, Hamlin I (2017). Generalised inhibitory impairment to appetitive cues: from alcoholic to non-alcoholic visual stimuli. Drug Alcohol Depend.

[45] van Deursen DS, Salemink E, Boendermaker WJ, Pronk T, Hofmann W, Wiers RW (2015). Executive functions and motivation as moderators of the relationship between automatic associations and alcohol use in problem drinkers seeking online help. Alcohol Clin Exp Res.

[46] Crane D, Garnett C, Michie S, West R, Brown J (2018). A smartphone app to reduce excessive alcohol consumption: identifying the effectiveness of intervention components in a factorial randomised control trial. Sci Rep.

[47] Zhang M, Ying J, Song G, Fung DS, Smith H (2018). Attention and cognitive bias modification apps: review of the literature and of commercially available apps. JMIR Mhealth Uhealth.

[48] Jones EB, Sharpe L (2017). Cognitive bias modification: a review of meta-analyses. J Affect Disord.

[49] Santarossa S, Kane D, Senn CY, Woodruff SJ (2018). Exploring the role of in-person components for online health behavior change interventions: can a digital person-to-person component suffice?. J Med Internet Res.

[50] Lumsden J, Skinner A, Woods A, Lawrence N, Munafò M (2016). The effects of gamelike features and test location on cognitive test performance and participant enjoyment. PeerJ.

[51] Johnson D, Deterding S, Kuhn K, Staneva A, Stoyanov S, Hides L (2016). Gamification for health and wellbeing: a systematic review of the literature. Internet Interv.

[52] Jacobus J, Taylor CT, Gray KM, Meredith LR, Porter AM, Li I, Castro N, Squeglia LM (2018). A multi-site proof-of-concept investigation of computerized approach-avoidance training in adolescent cannabis users. Drug Alcohol Depend.

[53] Lim CR, Barlas J, Ho RC (2018). The effects of temperament on depression according to the schema model: a scoping review. Int J Environ Res Public Health.

[54] Tran BX, McIntyre RS, Latkin CA, Phan HT, Vu GT, Nguyen HL, Gwee KK, Ho CS, Ho RC (2019). The current research landscape on the artificial intelligence application in the management of depressive disorders: a bibliometric analysis. Int J Environ Res Public Health.

