# Supplementary material for: Evidence Regarding Automatic Processing Computerized Tasks Designed For Health Interventions in Real-World Settings Among Adults: Systematic Scoping Review
Source: J Med Internet Res. 2020 Jul 29;22(7):e17915. doi: 10.2196/17915 (PMC7424486; doi:10.2196/17915)
Supplement: Multimedia Appendix 1 [file jmir_v22i7e17915_app1.docx]

**PubMed**

| **Automatic Processing**  **AND** | **Computerised Tasks**  **AND** | **Health Interventions**  **AND** | **Real-world**  **AND** | **Adult**  **AND** |
| --- | --- | --- | --- | --- |
| “Automatic process*”[tiab] OR “Implicit biases”[tiab] OR “automatic evaluation*”[tiab] OR “association learning”[tiab] OR “association learning”[mh] “Cognition”[tiab] OR “cognitive training”[tiab] OR “cognitive bias modification”[tiab] OR “motivation training”[tiab] OR “dual process*”[tiab] | “Computerised tasks”[tiab] OR “Computerized tasks”[tiab] “App-based[tiab] OR mobile technolog*”[tiab] OR “mobile app*”[tiab] OR “mobile applications”[mh] OR “software app*”[tiab] OR “electronic app*”[tiab] OR “health information systems”[mh] OR  “health information system*”[tiab] OR “telemedicine”[mh:noexp] OR “mobile health” [tiab] OR “mHealth”[tiab] OR “ telehealth”[tiab] OR “eHealth”[tiab] OR “computers/utilization”[mh] OR “serious games”[tiab] or “computer games”[tiab] | “Health Intervention*”[tiab] OR “health program*”[tiab] OR “preventative health”[tiab] OR “preventive health”[tiab] or “intervention*[tiab] | “Real world”[tiab] OR “Natural world”[tiab] OR “randomised controlled”[tiab} OR “non-randomised controlled”[tiab] OR “randomized controlled”[tiab} OR “non-randomized controlled”[tiab] | Adult[mh] OR “Older people”[tiab] OR “middle aged”[tiab] OR “young adult”[tiab] OR elderly[tiab] |

**Scopus**

| **Automatic Processing**  **AND** | **Computerised Tasks**  **AND** | **Health Interventions**  **AND** | **Real-world**  **AND** | **Adult**  **AND** |
| --- | --- | --- | --- | --- |
| {Automatic processing} OR {automatic processes} OR {Implicit biases} OR {implicit bias} OR {automatic evaluations} OR {automatic evaluation} OR {association learning} OR {Cognition} OR {cognitive training} OR {cognitive bias modification} OR {motivation training} OR {dual process} OR {dual processing} OR {dual processes} | {Computerized tasks} OR {Computerized tasks} OR {App-based} OR {mobile technology} OR {mobile app} OR {mobile applications} OR {mobile application} OR {software app} OR {software application} OR {electronic application} OR {electronic app} OR {health information systems} OR  {health information system} OR {telemedicine} OR {mobile health} OR {mHealth} OR { telehealth} OR {eHealth} OR {computers/utilization} OR {serious games} OR {computer games} | {Health Intervention} OR {health interventions} OR {health program} OR {health programs} OR {preventative health} OR {preventive health} OR {intervention}OR {interventions} | {Real world} OR {Natural world} OR {randomised controlled} OR {non-randomised controlled}OR {randomized controlled} OR {non-randomized controlled} | Adult OR {Older people} OR {middle aged} OR {young adult} OR {elderly} |

**Psychinfo**

| **Automatic Processing**  **AND** | **Computerised Tasks**  **AND** | **Health Interventions**  **AND** | **Real-world**  **AND** | **Adult**  **AND** |
| --- | --- | --- | --- | --- |
| Automatic process*.ti,ab OR Implicit biases*.ti,ab OR automatic evaluation*.ti,ab OR association learning.ti,ab OR Cognition.ti,ab OR cognitive training.ti,ab OR cognitive bias modification.ti,ab OR motivation training.ti,ab OR dual process*.ti,ab | Computeri#ed tasks.ti,ab OR App-based.ti,ab OR mobile technolog*.ti,ab OR mobile app*.ti,ab OR OR software app*.ti,ab OR electronic app*.ti,ab OR  health information system*.ti,ab OR telemedicine.ti,ab OR mobile health .ti,ab OR mHealth.ti,ab OR telehealth.ti,ab OR eHealth.ti,ab OR computers/utili#ation*.ti,ab OR serious game*.ti,ab or computer game*.ti,ab | Health Intervention*.ti,ab OR health program*.ti,ab OR preventative health.ti,ab OR preventive health.ti,ab or intervention*.ti,ab | Real world.ti,ab OR Natural world.ti,ab OR randomi#ed controlled.ti,ab OR non-randomi#ed controlled.ti,ab | Adult*.ti,ab OR Older people.ti,ab OR middle aged.ti,ab OR young adult*.ti,ab OR elderly.ti,ab |

**Embase**

| **Automatic Processing**  **AND** | **Computerised Tasks**  **AND** | **Health Interventions**  **AND** | **Real-world**  **AND** | **Adult**  **AND** |
| --- | --- | --- | --- | --- |
| automatic process* OR Implicit biases* OR automatic evaluation* OR association learning OR Cognition OR cognitive training OR cognitive bias modification OR motivation training OR dual process* | computerised task* OR app-based OR mobile technolog* OR mobile app* OR software app* OR electronic app* OR  health information system* OR telemedicine OR mobile health OR mHealth OR telehealth OR eHealth OR computers OR serious game* OR computer game* | health Intervention* OR health program* OR preventative health OR preventive health or intervention* | real world OR Natural world OR randomised controlled OR non-randomised controlled | adult* OR Older people OR middle aged OR young adult* OR elderly |

**CINAHL:**

| **Automatic Processing**  **AND** | **Computerised Tasks**  **AND** | **Health Interventions**  **AND** | **Real-world**  **AND** | **Adult**  **AND** |
| --- | --- | --- | --- | --- |
| Automatic process* OR Implicit biases* OR automatic evaluation* OR association learning OR cognition OR cognitive training OR cognitive bias modification OR motivation training OR dual process* | Computeri?ed task* OR app-based OR mobile technolog* OR ‘mobile app* OR software app* OR electronic app* OR  health information system* OR telemedicine OR mobile health OR mHealth OR telehealth OR eHealth OR computers/utili?ation* OR serious game* or computer game* | Health Intervention* OR health program* OR preventative health OR preventive health or intervention* | Real world OR Natural world OR randomi?ed controlled OR non-randomi?ed controlled | Adult* OR Older people OR middle aged OR young adult* OR elderly |

| **Automatic Processing**  **AND** | **Computerised Tasks**  **AND** | **Health Interventions**  **AND** | **Real-world**  **AND** | **Adult**  **AND** |
| --- | --- | --- | --- | --- |
| “Automatic process” OR “Implicit biases” OR “automatic evaluation” OR “association learning” OR Cognition OR “cognitive training” OR “cognitive bias modification” OR “motivation training” OR “dual process” | “Computerised task” OR “app-based” OR “mobile technology” OR “mobile app” OR “software app” OR “electronic app” OR  “health information system” OR telemedicine OR “mobile health” OR mHealth OR telehealth OR eHealth OR computers OR “serious game” or “computer game” | “Health Intervention” OR “health program” OR “preventative health” OR “preventive health” or intervention | “Real world” OR “Natural world” OR “randomised controlled” OR “non-randomised controlled” | Adult OR “Older people” OR “middle aged” OR “young adult” OR elderly |

**Cochrane:**
