# Supplementary material for: Evidence Regarding Automatic Processing Computerized Tasks Designed For Health Interventions in Real-World Settings Among Adults: Systematic Scoping Review
Source: J Med Internet Res. 2020 Jul 29;22(7):e17915. doi: 10.2196/17915 (PMC7424486; doi:10.2196/17915)
Supplement: Multimedia Appendix 2 [file jmir_v22i7e17915_app2.docx]

*Data Extraction Form*

**Reviewers Name:**

**Date of form completion:**

**Journal and ranking:**

***References to this Trial***

Check other references identified in searches. If there are further references to this trial (i.e. multiple publications for the same study) link the papers now & list below.

| Code each paper | References  Format: [Surname] [initial], (etc). [Title of paper]. [*Journal title*]. [Year of publication]; [volume] [(issue)]: [page numbers] |
| --- | --- |
| **A** |  |
| **B** |  |
| **C** |  |

***Methods:***

|  | Further details |
| --- | --- |
| Objective: *(Aim of the study as specified in paper)* |  |
| Design as specified in paper: *(i.e. RCT, how many arms?)* |  |
| Did the study design match what was specified? If not, why? |  |
| Year: |  |
| Country: |  |
| Study Site: *(Homes etc.)* |  |
| Methods of Analysis: *(Logistic regression, stem-and-leaf and box plots etc)* |  |
| Recruitment incentive: *(money, credits etc.)* |  |

***Participants:***

| Eligible for study (total n-value of whole study): |  | | |
| --- | --- | --- | --- |
|  | Further details  Intervention: Control: | | OR Other (usual care): |
| Randomised: (n-value for each arm of study randomised) |  |  |  |
| Completed: (participant completion n-value for each arm of study) |  |  |  |
| Age: (mean value &/or range for each arm) |  |  |  |
| Gender: (for each arm) |  |  |  |
| Recruitment: *(through what means flyers, website etc.)* |  | |  |
| Reasons for subject exclusion: |  | |  |
| Reasons for subject inclusion: |  | |  |

***Interventions:***

|  | Further details |
| --- | --- |
| Setting *(lab, home etc.):* |  |
| Duration of intervention: |  |
| Intervention delivered by: (researchers, company)  **If not stated can you deduce?* |  |
| Details of theory behind intervention: *(Dual-process models, gamification models, cognitive bias modification models etc.)* |  |

| Targeted intervention strategies: (provide brief description) |  |
| --- | --- |
| Cognitive bias modification |  |
| Automatic evaluations |  |
| Gamification |  |
| Engagement strategies |  |
| Other strategies (please specify) |  |
| Type of intervention: (tick all those that apply and provide brief descriptions): |  |
| Web-based |  |
| App-based |  |
| Smart-phone |  |
| Other digital means (please specify) |  |
| Type of control: Are any of the above interventions also in the control group? If so, describe: |  |
| Web-based |  |
| App-based |  |
| Smart-phone |  |
| Other digital means (please specify) |  |

***Outcomes:***

|  | Further details |
| --- | --- |
| List the pre specified primary outcomes and how they were measured: |  |
| List the pre specified secondary outcomes and how they were measured: |  |
| Follow-up:(baseline,post-int) |  |
| Additional Notes: |  |

***Data extraction***

| **Topics relevant to your review for Discussion** | **Reported in paper**  **(YES / NO)** | **Details** |
| --- | --- | --- |
| **Relevant studies and/or reviews identified in ref list** |  |  |
| **Automatic processing/automatic evaluations/implicit biases** |  |  |
| **Population group-adults** |  |  |
| **Computerised tasks/cognitive bias modification tasks** |  |  |
| **Health intervention** |  |  |
| **Effectiveness of intervention** |  |  |
| **Reasons of effectiveness/ or why it didn’t work** |  |  |
| **Real-world setting** |  |  |
| **Gaps in literature** |  |  |
