# Supplementary material for: Evidence Regarding Automatic Processing Computerized Tasks Designed For Health Interventions in Real-World Settings Among Adults: Systematic Scoping Review
Source: J Med Internet Res. 2020 Jul 29;22(7):e17915. doi: 10.2196/17915 (PMC7424486; doi:10.2196/17915)
Supplement: Multimedia Appendix 3 [file jmir_v22i7e17915_app3.docx]

| Reference | Condition targeted | Description of intervention | Measure | Main outcomes reported |
| --- | --- | --- | --- | --- |
| *Robinson 2017*[32] | Smoking | Attentional re-training-visual probe task | Attentional re-training-visual probe task | To investigate the effect of attentional retraining (AR) on attentional bias and smoking in African American smokers. AR administered on a mobile device reduced attentional bias in African American smokers and had mixed effects on smoking. |
| *Clarke 2015*[34] | Insomnia | Attentional bias modification-attentional probe task | Attentional bias modification-attentional probe task and behavioural measure | To assess whether targeted delivery of an ABM task during the pre-sleep period could reduce symptoms of insomnia and the cognitive symptoms of pre-sleep arousal. These results suggest that attentional bias modification targeting vigilance for sleep-related threat during the pre-sleep period has the capacity to reduce cognitive arousal and improve insomnia symptoms. |
| *Yang 201*7[33] | Social anxiety | Cognitive bias modification-attention bias modification (dot probe tasks) and interpretation bias | Dot-probe test  word sentence association paradigm | To compare the effectiveness of three different types of training programmes (cognitive bias modification-attention, CBM-A; cognitive bias modification-interpretation, CBM-I; attention and interpretation modification, AIM) administered via smart-phones by using a control condition (CC). The study supports the feasibility of delivering CBM-I via smartphones, but the effectiveness of CBM-A and AIM training via smartphones was limited. |
| *Fleming 2017*[36] | Self-esteem and negative attitudes that gay men may have towards homosexuality. | Evaluative conditioning task | Self-esteem implicit association test and sexual orientation implicit association test | To determine if evaluative conditioning deployed over the Internet could modify self-esteem and negative attitudes that gay men may have towards homosexuality. Internet-based EC did not produce significant effects in implicit or explicit self-directed attitudes. |
| *Deursen 2015*[37] | Alcohol abuse | Cognitive bias modification-consisting of three tasks: attentional bias retraining, alcohol/no-go training, and approach bias retraining. | Implicit association test | It was expected that executive functions (working memory, response inhibition) would moderate the relationship between automatic associations and alcohol use and that this effect would be stronger in individuals with strong motivation to change. Results provide partial support for the moderating role of motivation in the interplay between automatic processes and executive functions. |
| *De Voogd 2016*[14] | Anxiety and depression | Attentional bias modification-Visual search attention training | Dot-probe and visual search based  attentional training | To investigate the efficacy of multiple sessions of online attentional bias modification training to reduce attentional bias and symptoms of anxiety and depression, and to increase emotional resilience in youth. The study provided no support for the efficacy of online attentional bias modification training as a preventive intervention to reduce symptoms of anxiety or depression or to increase emotional resilience in youth. |
| *Enock 2014*[15] | Social anxiety | Attention bias modification-dot-probe training | Attention bias modification-dot-probe training | To test the efficacy of smart phone delivered CBM-A. No statistical significance between the two groups in reducing social anxiety was found. |
| *Monk 2017*[30] | Alcohol abuse | Evaluative conditioning- stop signal task | stop signal task-visual stimuli | The current study examined the hitherto untested assertion that the disinhibiting effects of alcohol-related stimuli might generalise to other appetitive liquid stimuli, but not to non-appetitive liquid stimuli. These findings suggest that decreases in inhibitory control in response to alcohol-related cues might generalise to other appetitive liquids, possibly due to evaluative conditioning. |
| *Weirs 2015*[20] | Alcohol abuse | Cognitive bias modification: attention control and approach bias re-training | Cognitive bias modification: attention control and approach bias re-training | Investigate whether different varieties of CBM (attention control training and approach-bias re-training) could be delivered successfully in a fully automated web-based way and whether these interventions would help self-selected problem drinkers to reduce their drinking. The general pattern of findings was that participants in all conditions reduced their drinking. |
| *Boendermaker 2016*[19] | Alcohol abuse | Gamified attentional bias modification-visual probe tasks | Alcohol Attention Control Training Program- pictorial alcohol-Stroop tasks  Assessment version of the VPT and another task that also measures attentional bias but is procedurally different, that  is, the visual search task (VST) | To prevent escalation of regular alcohol use into problematic use in university students through the use of a gamified cognitive bias modification game. The novel game-like approach proved insufficient to motivate young adults to train, in comparison with a regular CBM-A training. In fact, some aspects of motivation appeared to deteriorate rather than improve, suggesting that gamification can have drawbacks if not done optimally. |
| *Crane 2018*[29] | Alcohol abuse | Cognitive bias re-training | Not stated | To evaluate intervention components of an alcohol reduction app: Drink Less. The combination of enhanced Normative Feedback and Cognitive Bias Retraining and enhanced Self-monitoring and Feedback and Action Planning yielded improvements in alcohol-related outcomes after 4-weeks. |
| *Elfeddali 2016*[31] | Smoking | Attentional bias modification-visual probe tasks | Visual probe task | To assess the efficacy of a multiple-sessions Web-based Attentional Bias Modification (ABM) self-help intervention in smokers who made a quit-attempt. Web-based ABM training is ineffective in fostering cognitive bias reduction and continued smoking abstinence. |
| *Franklin 2016*[38] | Self-injurious thoughts and behaviours (suicide). | Therapeutic evaluative conditioning-game-like app | Affect misattribution procedure | The goal of this series of studies was to take initial steps toward developing an effective self-injurious thoughts and behaviours treatment that could easily be delivered on a very large scale. Two of the 3 studies showed that therapeutic evaluative conditioning impacted its intended treatment targets and that greater change in these targets was associated with greater self-injurious thoughts and behaviours reductions. |
| *McNulty 2017*[35] | Relationship satisfaction. | Evaluative conditioning | Evaluative-priming task | Examined whether directly altering affective associations involving a relationship partner through evaluative conditioning could lead to changes in relationship satisfaction. These results provide novel evidence for a mechanism of change in relationship satisfaction. |
